# Molecular characterization of serogroup 19 *Streptococcus pneumoniae* in the Czech Republic in the post-vaccine era

**DOI:** 10.1099/jmm.0.000765

**Published:** 2018-06-01

**Authors:** Helena Žemličková, Lucia Mališová, Petra Španělová, Vladislav Jakubů, Jana Kozáková, Martin Musílek, Matej Medvecký

**Affiliations:** ^1^​ Centre for Epidemiology and Microbiology, National Institute of Public Health, Prague, Czech Republic; ^2^​ Department of Clinical Microbiology, Faculty of Medicine and University Hospital, Charles University, Hradec Kralove, Czech Republic; ^3^​ Veterinary Research Institute, Brno, Czech Republic

**Keywords:** *Streptoccoccus pneumoniae*, MLST, WGS

## Abstract

**Purpose:**

The aim of this study was to characterize serogroup 19 isolates resistant to macrolides and/or penicillin found among pneumococci recovered from cases of invasive and respiratory tract disease in the Czech Republic in 2014.

**Methods:**

Pneumococcal isolates of serotypes 19A (*n*=26) and 19F (*n*=10) that were non-susceptible to penicillin and/or macrolides and had been collected in 2014 were analysed using multi-locus sequence typing (MLST). Four isolates representing the major clones were subjected to whole-genome sequencing (WGS).

**Results:**

The penicillin-susceptible macrolide-resistant isolates of serotype 19A were mainly associated with sequence type (ST) 416 belonging to clonal complex (CC) 199, and the penicillin-resistant isolates were of serotype 19F belonging to ST1464 (CC 320). WGS revealed the presence of pilus 1, in association with pilus 2, in serotype19F isolates belonging to CC 320. Another adhesin, pneumococcal serine-rich protein (PsrP), was only present in serotype 19A isolates of ST416. Analysis of the penicillin-binding proteins (PBPs) of serotype 19F penicillin-resistant isolates (ST1464 and ST271) performed on PBP1a, 2b and 2x identified a large number of mutations in comparison to the reference strain, R6. Both isolates contained a unique PBP profile; however, they were highly similar to PBP sequences of the Taiwan^19F^-14 reference strain. The *Pbp2b* sequences of both 19F isolates showed the lowest similarity to those of the Taiwan^19F^-14 strain (91 % similarity), while they were also found to be distantly related to each other (94 % similarity).

**Conclusions:**

WGS revealed specific virulence factors in antibiotic-resistant pneumococcal clones that spread rapidly in the post-vaccine era in the Czech Republic.

## Introduction

*Streptoccoccus pneumoniae* is an important pathogen of respiratory tract infections and a leading cause of bacteraemia and meningitis. The occurrence of *S. pneumoniae* that are resistant to betalactams and other groups of antibiotics complicates the effective treatment of pneumococcal infections [1]. It was predicted that the dissemination of antibiotic-resistant pneumococci would be targeted by the pneumococcal conjugate vaccines, as the serotypes included in the vaccines had accounted for the vast majority of penicillin non-susceptible serotypes [2]. After the implementation of nationwide pneumococcal vaccination for children, the incidence of invasive pneumococcal disease (IPD) has decreased among the vaccinated and, due to the herd effect, it has also decreased in the non-vaccinated population [3, 4]. Although a reduction in invasive disease has been reported, especially in cases caused by penicillin-resistant serotypes, the reduction of intermediately penicillin-resistant strains of vaccine serotypes has been compensated by increased intermediate resistance among non-vaccine serotypes [5–7].

The clone that has the highest impact on the spread of antibiotic resistance in the post-PCV7 era is Taiwan^19F^-14 [8–10]. This clone, which shows sequence type (ST) 236, was originally detected as having the 19F capsule, and was resistant to betalactams, macrolides and tetracyclines [11]. The surveys following the implementation of pneumococcal vaccination identified isolates belonging to this clone and showing serotype 19A in many countries in Asia and Europe, as well as in the United States [8, 12, 13]. Occasionally, 19A isolates expressing ST320, which is a double-locus variant of ST236, were found in the pre-vaccine era. After the introduction of PCV7, ST320 19A isolates have become the most prevalent multidrug-resistant genotype in the post-vaccine era in the United States and some European countries, and have continued to be identified in the PCV13 era [14–17]. In the Czech Republic, vaccination with PCV10 or PCV13 (covered by health insurance) has been included in the routine childhood immunization programme since January 2010. The use of PCV is voluntary and, according to data from the major health insurance company, 73.9 % of children up to 1 year of age had been vaccinated in 2014. In the post-PCV era, isolates of the 19A serotype that are non-susceptible to penicillin and/or to macrolides and tetracyclines have started to occur in carriage and respiratory samples and have also been found in IPD cases in all age cohorts [18]. The historical pre-vaccine antibiotic-resistant 19A isolates identified in the Czech Republic belonged to two clones, Hungary^19A^-6 and CSR^19A^-11 [19]. Isolates of the Taiwan^19F^-14 clone were not found in our environment in either serotype 19A or 19F isolates, which were mainly associated with ST423 [20]. Since the mid-1990s, serotype 9V, represented by the Spain^9V^-3 clone, has replaced serotype 19A (the Hungary^19A^-6 clone), and the total prevalence of serotype 19A declined dramatically [21]. It can be assumed that the implementation of vaccination against pneumococcus has affected the serotype-specific prevalence of pneumococci, including the antibiotic-resistant serotypes, but other factors that have been enhancing the spread of the antibiotic-resistant 19A serotype must be taken into account, as both currently used vaccines (with different 19A coverage) share the market almost equally, and the effect on the replacement by the 19A serotype is therefore limited.

The serotyping of antibiotic-resistant serotypes has shown the increasing prevalence of the 19A serotype following vaccination against pneumococci; however, this information is not sufficient to identify the origin of the strains. To establish the genetic background of the antibiotic-resistant 19A isolates, we used multi-locus sequence typing (MLST) to identify the clonal lineages associated with antibiotic resistance after the implementation of pneumococcal vaccination. The detailed characteristics of the representative isolates of the major serogroup 19 clones have been further identified by whole-genome sequencing (WGS).

## Methods

### Bacterial isolates

In total, 36 pneumococcal isolates of serotypes 19A (*n*=26) and 19F (*n*=10) that were non-susceptible to penicillin and/or macrolides and were collected in 2014 were analysed. Isolates from the blood and cerebrospinal fluid (*n*=16) were identified among all (*n*=306) invasive isolates referred to the National Institute of Public Health (NIPH) for antibiotic susceptibility testing and serotyping during national surveillance of IPD. Additional isolates (*n*=20) were obtained during the surveillance of antibiotic resistance in *S. pneumoniae* isolates from respiratory tract disease (sinusitis, acute otitis media and community-acquired pneumonia), where only isolates resistant to erythromycin (*n*=27) are referred to the NIPH.

All isolates were cultured on Columbia agar, and the species identification was confirmed using the deoxycholate test. The serotyping was performed by multiplex PCR using primers and conditions available at the Centers for Disease Control and Prevention (https://www.cdc.gov/streplab/pcr.html). Serotypes were confirmed by the Quellung reaction [22].

### Antibiotic susceptibility

The minimum inhibitory concentrations (MICs) were determined for penicillin (PEN), cefotaxime (CTX), tetracycline (TET), erythromycin (ERY), clindamycin (CLI), chloramphenicol (CHL) and trimethoprim/sulfamethoxazole (SXT) by the microdilution broth method [23]. The MICs were interpreted according to the criteria of the European Committee on Antimicrobial Susceptibility Testing (EUCAST; www.eucast.org). For epidemiological purposes, the microbiological breakpoints of low-level PEN resistance (MIC range from 0.12 to 2 µg m1^−1^) and high-level PEN resistance (MIC>2 µg m1^−1^) were used. The resistance to macrolides and tetracyclines was confirmed by PCR amplification of the respective genes. Resistance to macrolides was determined by PCR of internal fragments of the *mefA* and *ermB* genes according to the previously described protocols [24, 25]. PCR detection of tetracycline resistance was performed by amplification of the 1862-bp fragment from positions 21 to 1882 of the published sequence of the *tetM* gene [26].

### MLST typing

DNA was isolated according to the manufacturer's instructions using a commercial kit (NA2120, Sigma Aldrich) enriched with 250 U ml^−1^ of mutanolysin (M9901, Sigma Aldrich). Samples were typed according to a standard protocol [27]. Briefly, the internal fragments of seven housekeeping genes (*aroE*, *gdh*, *gki, recP, spi, xpt* and *ddl*) were amplified by PCR. The editing and alignment of all the sequences were performed using the publicly available *S. pneumoniae* MLST database (http://pubmlst.org/spneumoniae/), where new STs were also deposited. Clonal complexes were assigned using the eBURST algorithm (http://eburst.mlst.net). The allelic profiles of the STs of all the isolates were analysed using BioNumerics 7.6 software (Applied Maths, Ghent, East Flanders, Belgium).

### WGS library, Illumina sequencing

Four isolates representing the major clones were analysed. The genomic DNA of *S. pneumoniae* was extracted using the DNA-Sorb-B kit (Sacace Biotechnologies Srl, Como, Italy). The plasmids and chromosomes were sequenced using the Illumina MiSeq platform (Illumina Inc., San Diego, CA, USA). The reads were mapped using the Short Read Sequence Typing 2 (SRST2) tool (version 0.2.0) to assess the presence of virulence and antibiotic resistance genes [28]. The database of virulence genes was obtained from VFDB (http://www.mgc.ac.cn/VFs/), and the database distributed with SRST2 was used to search for resistance genes. The serotypes were determined by mapping reads to the reference *cps* locus sequences using PneumoCat [29]. The initial reads were quality trimmed using Trimmomatic [30] and assembled via the de Bruijn graph-based *de novo* assembler SPAdes [31]. The STs of the four *de novo* assembled genomes were confirmed on http://pubmlst.org/perl/bigsdb/bigsdb.pl?db=pubmlst_spneumoniae_seqdef&page=sequenceQuery. Further analyses of the assembled sequences were performed using Bionumerics 7.6. Gene annotations were carried out by the Bionumerics annotation application using the Taiwan^19F^-14 element (GenBank accession number CP000921) as the template sequence. Sequences of *pbp1a*, *pbp2b* and *pbp2x,* virulence and antibiotic resistance determinants of two 19A penicillin-susceptible and two 19F penicillin-resistant strains, were aligned to the corresponding sequences of *S. pneumoniae* R6 (accession number NC_003098.1) and compared in detail. The nucleotide sequences of the *pbp* genes were compared with the sequences of Taiwan^19F^-14 (GenBank accession number CP000921), Hungary^19A^-6 (GenBank accession number CP000936.1) and multidrug-resistant *S. pneumoniae* strain A026 (GenBank accession number CP006844) [32]. The mutations identified in the nucleotide sequence of *pbp2b* from the penicillin-resistant isolates were listed and compared with the protein sequence mutations reported by Contreras-Martel [33]. Pairwise clustering was processed by Bionumerics software using its default settings.

### Accession numbers

Nucleotide sequences representing each a different *pbp* type obtained in this study were submitted to GenBank. The nucleotide sequences of *pbp2x* and *pbp2b* (isolate 24238) and *pbp2x* and *pbp2b* (isolate 25137) were deposited in GenBank under accession numbers MF033177, MF033178, MF033179 and MF033180, respectively.

## Results

### Antibiotic susceptibility

In 2014, 306 invasive isolates of *S. pneumoniae* were examined at the National Institute of Public Health, Prague, Czech Republic. Of these, 16 (5.2 %) were non-susceptible to penicillin. Only three isolates belonging to serotype 19F showed high-level resistance to penicillin. The most common serotype among the isolates with low-level resistance (*n*=13) was serotype 19A (*n*=4; 30.8 %). Resistance to erythromycin was detected in 22 (7.2 %) isolates and was associated with serotype 19A (*n*=10; 45.4 %), followed by serotype 19F (*n*=4; 18.2 %). While serotype 19A was the second most common invasive serotype (*n*=25; 8.2 %), serotype 19F was far rarer (*n*=8; 2.6 %). Among the non-invasive respiratory isolates (*n*=27), resistance to erythromycin was linked with serotypes 19A (*n*=15; 55.5 %) and 19F (*n*=6; 22.2 %), and in almost half of the isolates (*n*=13; 48.1 %) it was combined with decreased susceptibility to penicillin. The median age of patients with serotype 19A was 42 (IQR 65.5–3) years, while it was 63.5 (IQR 70–60.5) years in serotype 19F-infected patients.

With the exception of two non-viable isolates, all serogroup 19 strains recovered in 2014 that were resistant to erythromycin and/or to penicillin (*n*=36) were further analysed.

Resistance to macrolides was confirmed in all (*n*=34) erythromycin-resistant isolates. All of them showed the MLS_B_ phenotype and possesed the *ermB* gene. Dual-macrolide resistance (*ermB* and *mefA* genotypes) was found predominantly in 19F isolates (see Table 1). Resistance to tetracycline, which is frequently associated with the MLS_B_, phenotype, was due to the presence of the *tetM* genes. All but one of the isolates (serotype 19A, ST 11197) were susceptible to chloramphenicol, and resistance to trimethoprim/sulfamethoxazole was particularly found in 19F isolates.

**Table 1.** Characteristics of serogroup 19 isolates (*n*=36) obtained in the Czech Republic in 2014

Isolates	Serotype	Source		MIC (µg ml^−1^)							ATB resistance genes	CC	ST	Allelic profile							
			Age	PEN	CTX	TET	ERY	CLI	CHL	SXT				*aroE*	*gdh*	*gki*	*recP*	*spi*	*xpt*	*ddl*	PMEN clone
24 679	19A	Blood	70	0.008	0.016	16.0	>4.0	4.0	2.0	0.25	*ermB, tetM*	199	416	1	13	14	4	17	51	14	Netherland^15B^-37/ST199
26 584	19A	Blood	60	0.008	0.016	16.0	>4.0	>4.0	4.0	0.25	*ermB, tetM*	199	416	1	13	14	4	17	51	14	
27 270	19A	CSF	36	0.016	0.016	16.0	>4.0	>4.0	2.0	0.25	*ermB, tetM*	199	416†	1	13	14	4	17	51	14	
27 374	19A	Ear	3	0.016	0.016	>16.0	>4.0	4.0	2.0	0.25	*ermB, tetM*	199	416	1	13	14	4	17	51	14	
27 389	19A	Ear	3	0.016	0.016	16.0	>4.0	>4.0	2.0	0.25	*ermB, tetM*	199	416	1	13	14	4	17	51	14	
27 417	19A	Blood	64	0.016	0.016	16.0	>4.0	4.0	4.0	0.25	*ermB, tetM*	199	416	1	13	14	4	17	51	14	
27 491	19A	Ear	3	<0.004	0.016	<0.125	>4.0	>4.0	2.0	0.25	ermB, tetM*	199	416†	1	13	14	4	17	51	14	
27 630	19A	Blood	68	0.016	0.016	16.0	>4.0	>4.0	2.0	0.25	*ermB, tetM*	199	416	1	13	14	4	17	51	14	
27 775	19A	Ear	Unknown	0.008	0.016	16.0	>4.0	>4.0	2.0	0.25	*ermB, tetM*	199	416	1	13	14	4	17	51	14	
27 776	19A	Ear	Unknown	0.016	0.016	16.0	>4.0	>4.0	2.0	0.25	*ermB, tetM*	199	416	1	13	14	4	17	51	14	
27 806	19A	Ear	0	0.016	0.016	16.0	>4.0	>4.0	2.0	0.25	*ermB, tetM*	199	416	1	13	14	4	17	51	14	
27 844	19A	Sputum	62	0.016	0.016	16.0	>4.0	>4.0	2.0	0.25	*ermB, tetM*	199	416	1	13	14	4	17	51	14	
27 854	19A	Ear	3	0.016	0.03	16.0	>4.0	>4.0	2.0	0.25	*ermB, tetM*	199	416	1	13	14	4	17	51	14	
27 897	19A	Nose	6	0.016	<0.004	>16.0	>4.0	>4.0	2.0	0.5	*ermB, tetM*	199	416	1	13	14	4	17	51	14	
28 206	19A	Blood	73	0.008	0.03	<0.125	>4.0	2.0	2.0	0.5	ermB, tetM*	199	416	1	13	14	4	17	51	14	
28 240	19A	Ear	0	0.008	0.03	16.0	>4.0	>4.0	2.0	0.5	*ermB, tetM*	199	416	1	13	14	4	17	51	14	
24 417	19A	Blood	48	0.125	0.06	<0.125	0.06	0.06	2.0	0.25		199	1756	8	20	14	4	17	4	14	
25 138	19A	Blood	71	0.008	0.03	>16.0	>4.0	>4.0	2.0	0.25	*ermB, tetM*	199	11 198	1	13	14	4	199	51	14	
25 137	19F	Blood	57	8.0	4.0	16.0	>4.0	>4.0	0.5	>4.0	*ermB, mefA, tetM*	320	271†	4	16	19	15	6	20	26	Taiwan^19F^-14/ST236
27 722	19A	Sputum	67	2.0	1.0	16.0	>4.0	>4.0	2.0	>4.0	*ermB, mefA, tetM*	320	1464	4	16	19	15	6	20	106	
24 238	19F	CSF	73	4.0	2.0	8.0	>4.0	>4.0	2.0	>4.0	*ermB, mefA, tetM*	320	1464†	4	16	19	15	6	20	106	
24 633	19F	Sputum	56	4.0	2.0	16.0	>4.0	>4.0	4.0	>4.0	*ermB, mefA, tetM*	320	1464	4	16	19	15	6	20	106	
24 870	19F	Sputum	65	4.0	2.0	16.0	>4.0	>4.0	2.0	>4.0	*ermB, mefA, tetM*	320	1464	4	16	19	15	6	20	106	
24 993	19F	Sputum	62	4.0	2.0	16.0	>4.0	>4.0	4.0	>4.0	*ermB, mefA, tetM*	320	1464	4	16	19	15	6	20	106	
27 721	19F	Sputum	60	2.0	1.0	16.0	>4.0	>4.0	2.0	>4.0	*ermB, mefA, tetM*	320	1464	4	16	19	15	6	20	106	
27 723	19F	Sputum	61	2.0	1.0	16.0	>4.0	>4.0	2.0	>4.0	*ermB, mefA, tetM*	320	1464	4	16	19	15	6	20	106	
27 992	19F	Blood	67	4.0	2.0	16.0	>4.0	>4.0	2.0	>4.0	*ermB, mefA, tetM*	320	1464	4	16	19	15	6	20	106	
28 205	19F	Sputum	73	4.0	2.0	16.0	>4.0	>4.0	2.0	>4.0	*ermB, mefA, tetM*	320	1464	4	16	19	15	6	20	106	
24 493	19A	Blood	21	0.25	0.25	16.0	>4.0	>4.0	2.0	0.125	*ermB, tetM*	230	230	12	19	2	17	6	22	14	Denmark^14^-32/ST230
28 258	19A	Sputum	69	0.5	0.125	16.0	>4.0	0.5	2.0	0.125	*ermB, tetM*	230	230	12	19	2	17	6	22	14	
27 314	19A	Blood	61	2.0	1.0	16.0	>4.0	0.125	2.0	1.0	*ermB, tetM*	230	276	2	19	2	17	6	22	14	
25 833	19A	Blood	60	0.5	0.25	>16.0	0.06	0.125	2.0	>4.0		230	2013	12	19	36	17	6	20	14	
27 227	19A	Ear	3	0.016	0.016	>16.0	>4.0	>4.0	2.0	0.25	*ermB, tetM*	193	3863	8	10	211	16	1	26	1	Greece^21^-30/ST193
29 469	19A	Blood	4	0.016	0.016	16.0	>4.0	>4.0	2.0	0.25	*ermB, tetM*	193	3863	8	10	211	16	1	26	1	
28 087	19F	Blood	79	0.125	0.06	16.0	4.0	>4.0	4.0	0.5	*ermB, tetM*	177	179	7	14	40	12	1	1	14	Portugal^19F^-21/ST177
27 102	19A	Ear	5	<0.004	0.008	>16.0	>4.0	0.125	16.0	4.0	*ermB, tetM*	66	11 197	1	5	41	5	10	616	8	

PEN, penicilin; CTX, cefotaxime; TET, tetracycline; ERY, erytromycin; CLI, clindamycin; CHL, chloramphenicol; SXT, trimethoprim/sulfamethoxazole; CC, clonal complex; ST, sequence type; PMEN, Pneumococcal Molecular Epidemiology Network.

New sequence types are shown in bold.

*Isolates were found to be phenotypically susceptible due to the deletion of a 26-bp-long segment (from nucleotide position 16 298 to 16323) of the *tetM* gene.

†Isolates included in the whole-genome sequencing.

### MLST analysis

MLST analysis revealed 11 different STs among serogroup 19 (see Table 1). eBURST analysis using the whole MLST database categorized isolates into six CCs. The largest CC, including 18 of the 36 isolates (50 %), was represented by ST416 (*n*=16), ST11198 and ST1756. ST416 is a subgroup founder in CC199 and is related to the Netherlands^15B^-37 clone. All isolates within this CC belonged to serotype 19A (18 of 26 isolates; 69.2 %) The second major CC (CC320), which comprised 10 of the 36 isolates (27.8 %), was represented by ST1464 (*n*=9) and ST271; both are single-locus variants of ST236, the representative of the Taiwan^19F^-14 clone. This clone was mainly associated with serotype 19F (9 out of 10 isolates; 90 %), while 1 ST1464 isolate was determined to be in serotype 19A. The other minor CCs were CC230, which is related to the Denmark^14^-32 clone (four isolates of serotype 19A); CC193, which is related to Greece^21^-30 (two serotype 19A isolates); CC177, which is related to Portugal^19F^-21 (a 19F isolate); and CC66 (a 19A isolate).

The susceptibility profile was highly consistent with the corresponding CC. With the exception of one ST1756 isolate, CC199 was represented by penicillin-susceptible, but erythromycin-, clindamycin- and tetracycline-resistant isolates. By contrast, all of the CC320 isolates showed high-level penicillin resistance, the MLS_B_ phenotype (due to the double-macrolide resistance mechanism) and resistance to tetracycline and trimethoprim/sulfamethoxazole. Isolates with low-level penicillin resistance were found in two minor CCs: CC230 and CC177.

### WGS

#### Virulence genes

Four representative isolates of two major clones (CC199 represented by ST416 serotype 19A isolates and CC320 represented by ST271 and ST320 serotype 19F isolates) were studied by WGS. To determine the presence of virulence factors, raw sequence reads were mapped against a virulence gene database. The absence of a gene was confirmed by examining the annotated sequence using blast. Apart from the genes involved in capsule biosynthesis, genes encoding virulence factors covering all four main groups of surface proteins [lipoproteins (LPs), leucin–proline–any amino acid–threonine–glycine (LPXTG) proteins, choline-binding proteins (CBPs) and non-classical surface proteins (NCPs)] were found in all of the isolates [34]. Of the 16 described choline-binding proteins, all of the isolates lacked *CbpI* and *CbpM*, which have been shown to interact with elastin [35]. Of the 18 LPXTG proteins described, genes coding for metalloprotease ZmpC and MucB were also missing in all of the isolates; moreover, both 19F isolates lacked the *psrP* gene encoding pneumococcal serine-rich protein (PsrP), which was previously demonstrated to play a role in lung-cell attachment and the invasion of epithelial cells [36]. All of the isolates contained the *RlrA* islet encoding pilus 1, and CC320 carried pilus 1 in association with pilus 2.

#### Antibiotic resistance

The genome contained two large transposons. The first one, *Tn*6002, showing 99.99 % similarity to the *Tn*6002 complete sequence (GenBank accession number AY898750.1), was found in 19A isolates (CC199). This transposon carried two antibiotic resistance genes: *tetM* and *ermB*. However, one 19A isolate (27491) carrying the *tetM* gene was found to be phenotypically susceptible due to the deletion of a 26-bp-long segment (spanning from nucleotide position 16 298 to 16 323 of the *tetM* gene), leading to a frameshift mutation. The other transposon was identified as *Tn*2010, showing 99.99 % similarity to the *Tn*2010 complete sequence (GenBank accession number AB426620.1). This transposon has been found in both 19F isolates (CC320), and aside from *tetM* and *ermB*, it also carries the *mefE*/*mel* operon.

#### Comparison of PBP genes

**Fig. 1.**
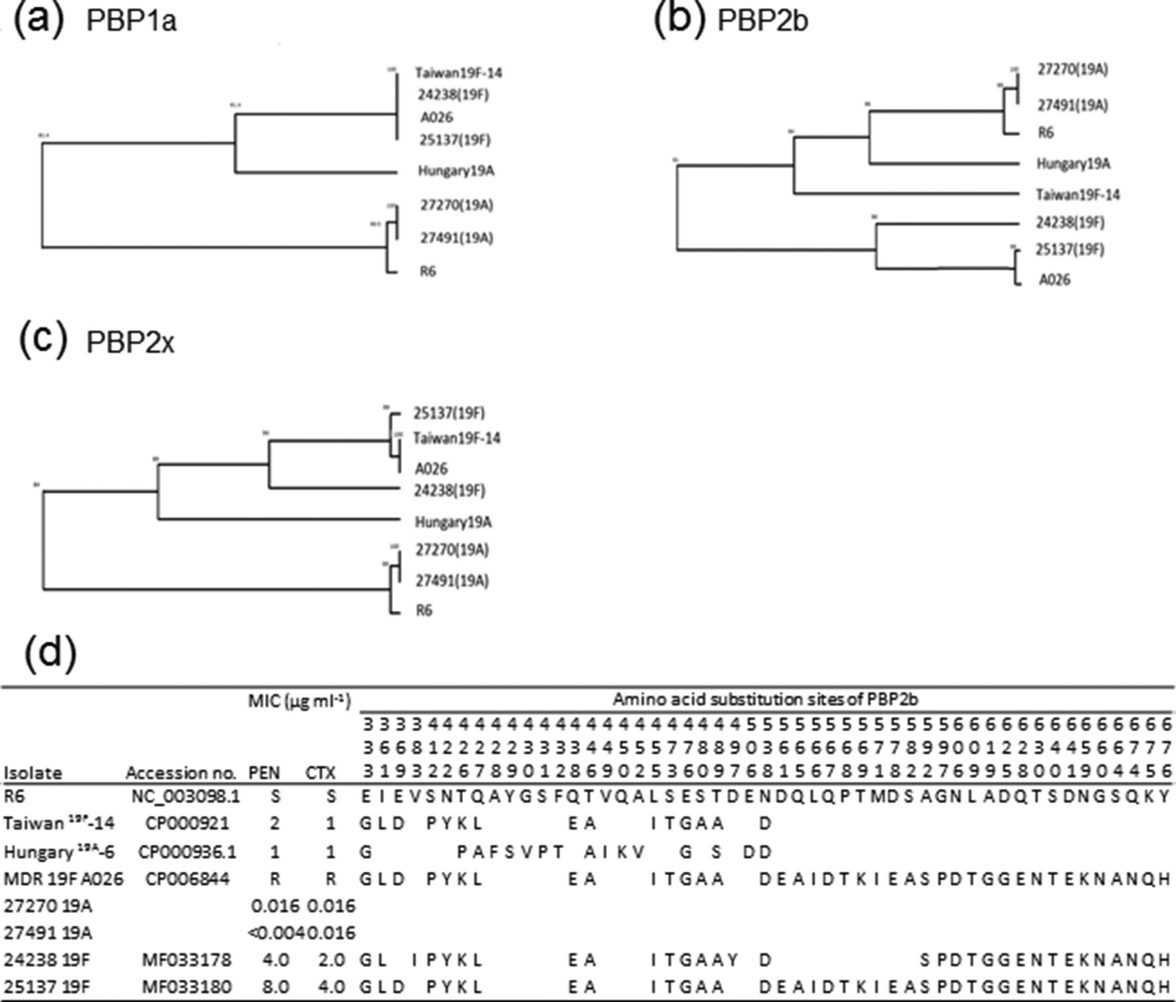
Genetic relationship of PBP1a, PBP2b and PBP2x amino acid sequences. (a–c) Pair-wise alignments generated by BioNumerics 7.6. showing the relatedness of *pbp1a* (a), *pbp2b* (b) and *pbp2x* (c) nucleotide sequences among penicillin-susceptible R6, multidrug-resistant 19F A026, Taiwan^19F^-14, Hungary^19A^-6 and 19A/19F isolates. (d) Amino acid substitution sites of PBP2b. The sequences were aligned according to the corresponding amino acid sequences of R6. PEN, penicillin; CTX, cefotaxime; S, susceptible; R, resistant.

The PBP sequences of *pbp2x*, *pbp2b* and *pbp1a* of penicillin-susceptible 19A isolates were highly identical (99.0 to 100 % similarity) to those of the R6 strain (see Fig. 1). The *Pbp* sequences of penicillin-resistant 19F isolates were additionally compared to the corresponding sequences of the Taiwan^19F^-14 and Hungary^19A^-6 reference strains and the multidrug-resistant A026 pneumococcal strain (GenBank accession number CP006844). The *Pbp1a* sequences of the two 19F isolates were identical. Moreover, they were 100 % similar to the *pbp1a* sequence of the Taiwan^19F^-14 reference strain. The *Pbp2x* sequence of the 25 137 isolate was also highly similar to the *pbp2x* sequence of the Taiwan^19F^-14 reference strain (99 % similarity), but differed from that of the other 19F isolate (94 % similarity). The PBP2x of the 25 137 isolate (ST271) possessed the unique amino acid (aa) substitutions M339F, E378A, M400T and Y595F, which are distinct from those in Taiwan^19F^-14 but identical to those reported in the ST271 clinical isolates from Hong Kong [37]. Isolate 24 238 (ST1464) possessed an additional five aa substitutions (P268T, D278N, N501K, K505E and A507T) compared to Taiwan^19F^-14, but none of them has yet been identified as playing a role in resistance to betalactams. Compared to the ST271 isolate, ST1464 lacks three mutations (M339F, M400T and Y595F) associated with cefotaxime resistance [38]. Both isolates contained mutations within the S337TMK motif: T338A in ST1464 and T338A plus M339F in the ST271 isolate. The *Pbp2b* sequences of both 19F isolates showed the lowest similarity to those of the Taiwan^19F^-14 strain (91 % similarity), while they were also distantly related to each other (94 % similarity). Comparison of the *pbp2b* sequences with the R6 sequence revealed the presence of different mosaic blocks in the two 19F isolates. The aa sequence of the 25 137 isolate (ST271) was identical to the sequences of the ST271 isolates of the 19F serotype from Hong Kong. The aa sequence of the other isolate (24238, ST1464) contained relatively short mosaic blocks at positions 333 to 538, which is highly similar to the corresponding Taiwan^19F^-14 reference aa sequence, but differed from ST271 in three substitutions. The ST1464 isolate possessed two additional mutations (V383I and D497Y) but lacked the E369D mutation conferring high-level resistance to cefotaxime. At positions 561 to 582, the isolates differed in nine aa substitutions (absent in ST1464); however, at positions 592 to 676, the isolates were identical and possessed 16 aa changes that have been reported before in resistant Polish isolates of the Poland^23F^-16 clone and in multidrug-resistant isolates of serogroup 19 from Hong Kong [37, 39].

## Discussion

Despite the relatively stable low prevalence of antibiotic resistance in invasive pneumococci, an increase in antibiotic-resistant serotype 19A has been observed in the Czech Republic. MLST analysis identified ST416 as the dominant emerging genotype, which was in contrast to previous studies that reported a post-vaccine clonal expansion of the 19A capsular variant of the Taiwan^19F^-14 clone, but in agreement with findings from other European countries, where clonal expansion of ST416 has been observed [9, 40–42]. It is estimated that the successful spread of serotype 19A is due to its genetic diversity, and various lineages of serotype 19A have emerged in the post-vaccine era [9]. The main drivers of the increasing prevalence of serotype 19A are antibiotic selection pressure and a high propensity for capsular switching. The pathogenicity of pneumococci has been attributed to various structures, most of which are located on its surface [43]. Numerous *in vitro* studies have identified different pneumococcal proteins as possible protein vaccine candidates [44–46]. Pneumococcal surface proteins are involved in bacterial fitness and pathogen–host interactions. Pneumococci are highly plastic bacteria, as they are able to adapt the expression of certain virulence factors to host niches during various stages of infection [47]. However, the prevalence of some of those surface proteins seems to be clonally related. It has been suggested that the presence of pilus 1 promotes adherence and thereby increases the competitive capacity of piliated strains [48]. Previousr studies have also provided evidence for an association between antibiotic-resistant clones and the presence of pilus 1 [48–51]. In our study, all of the WGS-analysed isolates carried pilus 1. The presence of pilus 1 in 19A isolates of ST416 is of interest, since group founder ST199 isolates are pilus-negative. It is likely that the acquisition of pilus 1 could promote a clonal expansion of this genotype to a significant degree. On the other hand, another adhesin, PsrP, was only present in 19A isolates of ST416. PrsP, which acts as the lung cell adhesin, was found to be significantly associated with serotypes with higher invasive disease potential (including the 19A serotype), and it was hypothesized that PsrP is most probably linked to enhanced virulence of those serotypes [52, 53]. Selva *et al*. have shown a negative correlation of the simultaneous prevalence of PsrP and pilus 1, suggesting that the production of extremely large proteins might be metabolically expensive and that individual strains either do not support the expression of both proteins, or the function of both adhesins is redundant [36]. However, based on the results of our study, it seems that both virulence factors act synergistically and jointly increase the fitness of the ST416 clonal lineage, which constituted at least 40 % of the serotype 19A population among IPD in 2014. Moreover, the overall prevalence of the 19A serotype increased from 2.7 % in 2010 to 9.9 % in 2015, with 19A being the second most frequently reported invasive serotype in the Czech Republic [54]. A tentative explanation is that the clonal expansion of 19A ST416/CC199, which is becoming more common than 19F ST1464/CC320, is due to the contribution of the co-presence of several serotype-independent factors, which makes a capsular type more prone to occupy the niche left by PCV10 serotypes.

The non-susceptibility to betalactams was mainly associated with serotype 19F of CC320, represented by its single locus variants (SLVs), ST1464 and ST271. These SLVs differed from ST320 in the *ddl* gene, which is highly variable in penicillin-resistant isolates due to the proximity of the *pbp2b* gene, which is affected by interspecies recombinational exchanges driven by penicillin usage [55]. Penicillin non-susceptibility in pneumococci is mediated by the recombination of *pbp* genes (namely *pbp1a*, *pbp2b* and *pbp2x*) leading to aa substitutions at or close to the active site of the transpeptidase domains [56, 57]. The development of penicillin resistance in pneumococci is driven by gene transfer events from related species, especially *Streptococcus mitis* or *Streptococcus oralis* [58, 59]. PBP2x and PBP2b are considered to be primary resistance determinants conferring low-level resistance, and an alteration of those PBPs is a prerequisite for the emergence of high-level resistance, together with a concomitant alteration of PBP1a [60]. Although both of the two penicillin-resistant isolates had a unique PBP profile, transpeptidase domains that were identical or related to those previously reported in pneumococci were detected in both isolates [33, 37, 39, 61]. The PBP1a sequence was identical to that of the Taiwan^19F^-14 strain. Moreover, the same PBP1a aa sequences have been described previously in clinical isolates of serogroup 19 (ST271 and ST320) [37]. It seems that this PBP1a variant is a rather stable characteristic of the Taiwan^19F^-14 clone, contributing to its typical phenotype. The PBP2x sequence of the ST271 isolate has also been identified in Hong Kong isolates of the corresponding serotype and ST [37]. The aa substitutions in PBP2x, such as T338A, M339F and M400T, were found to be responsible for cefotaxime resistance [38]. The presence of these aa substitutions, together with additional changes (E378A and Y595F), are important for the emergence of high-level resistance to cefotaxime [37, 62]. The presence of mutations was in line with the observed high level of resistance to cefotaxime in the isolate of ST271, while the ST1464 isolate, where three of these aa changes (M339F, M400T and Y595F) were absent, showed low-level resistance (cefotaxime MIC of 2 µg ml^−1^). Both ST271 and ST1464 possessed aa substitutions of PBP2b involved in enzyme activity (T446A, A619G and Q628E). The *Pbp2b* sequences contained aa substitutions that were absent in Taiwan^19F^-14. Whereas ST271 was identical to the ST271 isolates of the 19F serotype reported in Hong Kong, ST1464 lacked 12 aa substitutions inside a block identified previously in the resistant *S. mitis* strain B22 (EMBL accession number AY187721). The patterns of the two *pbp2b* were highly similar to those of variants IV (ST271) and V (ST1464) of *pbp2b*, respectively, shown in Polish isolates of the Poland^23F^-16 clone [39]. Our results demonstrate the diversity of *pbp* genes, which occurs even at the subclone level, and corroborate the necessity for a detailed analysis of *pbp* polymorphism.

In conclusion, after pneumococcal vaccination, new prevailing clones of serotypes 19A and 19F have been identified among antibiotic-resistant invasive pneumococci in the Czech Republic. The success of those clones is probably linked to the presence of specific characteristics (namely pilus 1), but other factors, such as post-vaccine serotype replacement or antibiotic consumption, may have favoured their further spread.
