# Corrigendum: Molecular characterization of serogroup 19 *Streptococcus pneumoniae* in the Czech Republic in the post-vaccine era

**DOI:** 10.1099/jmm.0.000795

**Published:** 2018-06-29

**Authors:** Helena Žemličková, Lucia Mališová, Petra Španělová, Vladislav Jakubů, Jana Kozáková, Martin Musílek, Matej Medvecký, Costas C. Papagiannitsis

**Affiliations:** ^1^​ Centre for Epidemiology and Microbiology, National Institute of Public Health, Prague, Czech Republic; ^2^​ Department of Clinical Microbiology, Faculty of Medicine and University Hospital, Charles University, Hradec Kralove, Czech Republic; ^3^​ Veterinary Research Institute, Brno, Czech Republic; ^4^​ Biomedical Center, Faculty of Medicine in Pilsen, Charles University, Pilsen, Czech Republic

**Keywords:** Corrigenda, WGS, *Streptoccoccus pneumoniae*, MLST

There was an error in the author list in the published article. The corrected list is as folllows:

Helena Žemličková; Centre for Epidemiology and Microbiology, National Institute of Public Health, Prague, Czech Republic; Department of Clinical Microbiology, Faculty of Medicine and University Hospital, Charles University, Hradec Kralove, Czech Republic;

Lucia Mališová; Centre for Epidemiology and Microbiology, National Institute of Public Health, Prague, Czech Republic;

Petra Španělová; Centre for Epidemiology and Microbiology, National Institute of Public Health, Prague, Czech Republic;

Vladislav Jakubů; Centre for Epidemiology and Microbiology, National Institute of Public Health, Prague, Czech Republic;

Jana Kozáková; Centre for Epidemiology and Microbiology, National Institute of Public Health, Prague, Czech Republic;

Martin Musílek; Centre for Epidemiology and Microbiology, National Institute of Public Health, Prague, Czech Republic;

Matej Medvecký; Veterinary Research Institute, Brno, Czech Republic.

Costas C. Papagiannitsis; Biomedical Center, Faculty of Medicine in Pilsen, Charles University, Pilsen, Czech Republic.

The author apologizes for any inconvenience caused.

